# Cardiometabolic multimorbidity may identify a more severe subset of rheumatoid arthritis, results from a “real-life” study

**DOI:** 10.1097/MD.0000000000033362

**Published:** 2022-04-07

**Authors:** Piero Ruscitti, Claudia Di Muzio, Alessandro Conforti, Ilenia Di Cola, Viktoriya Pavlych, Luca Navarini, Damiano Currado, Alice Biaggi, Stefano Di Donato, Annalisa Marino, Sebastiano Lorusso, Francesco Ursini, Roberto Giacomelli, Paola Cipriani

**Affiliations:** a Department of Biotechnological and Applied Clinical Sciences, University of L’Aquila, L’Aquila, Italy; b Rheumatology and Immunology Unit, Department of Medicine, University of Rome “Campus Biomedico”, Rome, Italy; c Medicine and Rheumatology Unit, IRCCS Istituto Ortopedico Rizzoli, Bologna, Italy; d Department of Biomedical and Neuromotor Sciences (DIBINEM), Alma Mater Studiorum University of Bologna, Bologna, Italy.

**Keywords:** cardiometabolic comorbidity, “difficult to treat” arthritis, rheumatoid arthritis

## Abstract

This “real-life” cross-sectional study has been designed to describe disease features of rheumatoid arthritis (RA) participants affected by cardiometabolic multimorbidity than those without. Our purpose was also the identification of possible associations between these cardiometabolic diseases and RA clinical characteristics. Consecutive RA participants with and without cardiometabolic multimorbidity were assessed and their clinical characteristics were recorded. Participants were grouped and compared by the presence or not of cardiometabolic multimorbidity (defined as ≥ 2 out of 3 cardiovascular risk factors including hypertension, dyslipidemia, and type 2 diabetes). The possible influence of cardiometabolic multimorbidity on RA features of poor prognosis was assessed. The positivity of anti-citrullinated protein antibodies, presence of extra-articular manifestations, lack of clinical remission, and biologic Disease-Modifying anti-Rheumatic Drugs (bDMARDs) failure were considered as RA features of poor prognosis. In the present evaluation, 757 consecutive RA participants were evaluated. Among them, 13.5% showed cardiometabolic multimorbidity. These were older (*P* < .001) and characterized by a longer disease duration (*P* = .023). They were more often affected by extra-articular manifestations (*P* = .029) and frequently displayed smoking habit (*P* = .003). A lower percentage of these patients was in clinical remission (*P* = .048), and they showed a more frequent history of bDMARD failure (*P* < .001). Regression models showed that cardiometabolic multimorbidity was significantly correlated with RA features of disease severity. They were predictors of anti-citrullinated protein antibodies positivity, of extra-articular manifestations, and of lack of clinical remission, in both univariate and multivariate analyses. Cardiometabolic multimorbidity was significantly associated with a history of bDMARD failure. We described disease features of RA participants with cardiometabolic multimorbidity, identifying a possible more difficult to treat subset, which may need a new management approach to achieve the treatment goal.

## 1. Introduction

Rheumatoid arthritis (RA) is a chronic inflammatory disease mainly affecting the joints which is associated with an increased morbidity and mortality than general population.^[1]^ In this context, a close relationship between RA and cardiovascular disease is reported in contributing to this enhanced risk of premature death.^[2]^ This typical clinical phenotype results from the synergy between the rheumatoid inflammatory process and the cardiovascular (CV) “traditional” risk factors. The latter result to be more prevalent and incident in RA.^[3]^ The presence of cardiometabolic multimorbidity, including dyslipidemia, type 2 diabetes (T2D), and high blood pressure (HBP), may represent a considerable burden in these patients.^[4]^ Interestingly, these comorbidities may share common pathogenic mechanisms, often clustering together.^[5]^ In fact, the assessment of a single comorbidity may fail to account for the complex interrelationships of multiple cardiometabolic conditions. Thus, the identification of clusters of this cardiometabolic multimorbidity has the potential to be more useful in clinical settings.^[6]^ Defining patterns of cardiometabolic multimorbidity may help to provide better understanding of how these features may affect the clinical picture of patients with RA. Moreover, the identification of multimorbidity patterns may facilitate the discovery of shared disease mechanisms or common therapeutic targets via translational research as well as the risk stratification of a population.^[7]^ In addition, shifting the focus to cardiometabolic multimorbidity has been recently emphasized over the need to define a metabolic syndrome. In fact, insights on clustering patterns in RA are limited to the metabolic syndrome and do not include broader cardiovascular risk factor clusters.^[2–5]^ A recent body of evidence also suggests that the cardiometabolic multimorbidity may be linked to RA by common pathogenic inflammatory mediators playing a critical role in metabolic dysregulation and accelerated atherosclerosis.^[5]^ It is also possible to hypothesize that the presence of such comorbidities may also contribute to increasing the pro-inflammatory burden and the hypercoagulable state of RA patients.^[8]^ Therefore, these patients with cardiometabolic multimorbidity may differ in their clinical presentation as well as in prognosis identifying a possible RA subset more difficult to treat. Coexisting cardiometabolic risk factors may either be the expression of a common pathogenic soil or cooperate with each other to predispose patients with RA to progress towards possible more severe clinical scenarios.^[8,9]^ In addition, in this context, it must be pointed out that the evidence deriving from randomized clinical trials does not entirely clarify this issue. In fact, given strict enrollment criteria the participants of these studies could not fully mirror the “real-life” situation.^[10]^ Although randomized clinical trials may provide an unbiased estimate of the comparative efficacy between patients in both treated and control groups, trial populations are often not fully representative of the patients encountered in clinical practice.^[11]^ Therefore, “real-life” studies may give relevant insights in a more heterogenous clinical setting, where many patients may have multiple comorbidities or other clinical features influencing their management.^[12,13]^

On these bases, we aimed to describe disease features of RA participants with cardiometabolic multimorbidity than those without in a “real-life” cohort. We also assessed the possible predictive role of the presence of cardiometabolic multimorbidity on RA features of poor prognosis.

## 2. Methods

### 2.1. Study design

**Figure 1.**
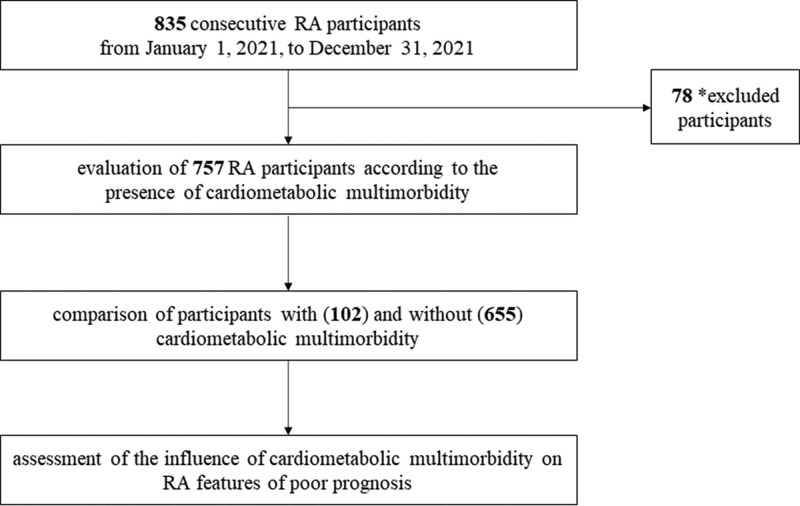
Study design. (*) Participants were excluded if their clinical records had significant meaningful missing data. Specifically, participants with 1 or more missing data in the main outcomes were removed from the analyses.

This cross-sectional observational study has been designed to describe the disease features of participants with RA affected by cardiometabolic multimorbidity than those without. From January 1, 2021, to December 31, 2021, consecutive participants with RA, admitted to Rheumatology Units of University of L’Aquila, L’Aquila and of University of Campus Biomedico of Rome, Rome were assessed, as detailed in Figure 1. The local Ethics Committee (*Comitato Etico Azienda Sanitaria Locale 1 Avezzano/Sulmona/L’Aquila, L’Aquila*, Italy; protocol number 000331/17) approved the study, which was performed according to the Good Clinical Practice guidelines and the Declaration of Helsinki. In reporting the results, we followed the STROBE guidelines.

### 2.2. Settings

Participants were selected among those attending Rheumatologic Units Rheumatology Units of University of L’Aquila, L’Aquila and of University of Campus Biomedico of Rome, Rome. These units were characterized by experience in management of RA as well as in observational studies and by high-volume outpatient clinics. Data of participants were recorded during the scheduled visits.

### 2.3. Participants

From January 1, 2021, to December 31, 2021, participants with RA were assessed. All participants fulfilled 2010 ACR/European Society of Rheumatology criteria for RA^[14]^; whereas those not fulfilling these criteria were not considered in this study.

### 2.4. Variables to be assessed

The participant was defined as having cardiometabolic multimorbidity if affected by 2 or 3 among HBP, T2D, and/or dyslipidaemia. HPB, T2D, and dyslipidaemia were defined as previously performed.^[4,9]^ We used this approach since cardiometabolic multimorbidity (≥2 of 3 risk factors) may be considered as a risk factor which may differ from a single cardiometabolic condition. This approach has been largely used in different settings from general population to chronic diseases to better investigate the impact of cardiometabolic multimorbidity on patient clinical picture.^[15–19]^ HPB was defined when participant blood pressure was consistently measured > 130 mm Hg for systolic and > 80 mm Hg for diastolic, according to the data in clinical charts, and/or the need of antihypertensive drugs.^[20]^ Participants was defined as having T2D if fasting plasma glucose ≥ 126 mg/dL in 2 different evaluations, according to the data in clinical charts, or in the presence of classic symptoms of hyperglycemia or hyperglycemic crisis with a random plasma glucose 200 mg/dL (11.1 mmol/L), or if antidiabetic therapies were administered.^[21]^ Considering the difficulties in assessing the values of lipid in RA and the so-called “lipid-paradox,”^[22,23]^ dyslipidemia was defined when drugs lowering the blood lipid levels were administered. Clinical atherosclerosis was defined as the presence of one of the following: myocardial infarction, congestive heart failure, cerebrovascular disease including transitory ischemic attack and/or stroke and clinically relevant peripheral artery disease, as previously performed.^[9,24]^ The researchers verified the presence of such comorbidities reviewing the clinical charts, by interview, and extensive medical examinations of participants.^[25]^ Among other “traditional” CV risk factors, gender, age, body mass index (BMI), and smoking habit were also recorded. BMI was calculated according to the standard formula weight (kg)/height (m)^2^. However, the obesity was not included in the definition of cardiometabolic multimorbidity considering the impaired predictive values of BMI on CV features in RA due to the “obesity paradox.”^[26,27]^

More in general, in our cohort, we defined the presence of other comorbidities at the time of assessment as coexisting medical conditions distinct from the principal diagnosis for which the participant was enrolled in this study.^[28]^ The presence of rheumatoid factor (RF), anti-citrullinated peptide antibodies (ACPA), extra-articular features,^[29]^ disease duration, disease activity, and remission were reported. Extra-articular features were defined as reported in a previous study.^[29]^ The following extra-articular manifestations were assessed: pericarditis, pleuritis, Felty’s syndrome, major cutaneous vasculitis, neuropathy, scleritis, episcleritis or retinal vasculitis, glomerulonephritis, vasculitis affecting other organs, amyloidosis, keratoconjunctivitis sicca, xerostomia, secondary Sjögren’s syndrome, pulmonary fibrosis, bronchiolitis obliterans organizing pneumonia, cervical myelopathy, subcutaneous rheumatoid nodules, rheumatoid nodules in other locations.^[29]^ Disease activity score including 28 joints was used to assess the disease activity and the remission state (disease activity score including 28 joints < 2.6). The administered therapeutic strategies were recorded, including conventional synthetic - and biologic disease-modifying antirheumatic drugs (bDMARDs). The participants, who underwent a sequential treatment with bDMARDs, were included in the category as failure of such drugs. As failure of bDMARDs, we defined participants who changed previous bDMARDs due to inefficacy, either primary or secondary, adverse events, contraindications, or intolerance, as reported in the clinical charts of the patients. Considering the specific design of our study, we did not stratify the failure of bDMARDs according to causes of discontinuation. Glucocorticoids therapy was codified in categories, high dosage, and low dosage.^[30]^ In this work, we also aimed to explore a clinical risk profile of patients with cardiometabolic multimorbidity in predicting some selected specific RA features associated with poor prognosis and a more difficult management. These characteristics were selected according to available literature of clinical features related to a more difficult to treat disease.^[31,32]^ Therefore, as also suggested from European Society of Rheumatology recommendations for management of RA patients,^[33]^ ACPA positivity, presence of extra-articular manifestations, lack of clinical remission, and bDMARD failure were considered as markers of poor prognosis.

### 2.5. Sample size and bias

No specific sample size has been estimated since a “real-life” evaluation of our study. Possible biases were minimized by a careful definition of each variable. Furthermore, participants with significant meaningful missing data were excluded. Specifically, participants with 1 or more missing data in the main outcomes were removed from the analyses.

### 2.6. Statistics

Statistics firstly provided descriptive analysis of the collected data. Continuous variables which were normally distributed were expressed as mean ± standard deviation or median and interquartile range, according to their distribution. Categorical variables were expressed as percentage of participants. Participants with and without cardiometabolic multimorbidity were grouped and compared by either parametric or nonparametric t-tests for continuous variables and Chi-squared test for categorical ones. After that, univariate and multivariate logistic regression models were built to assess the possible influence of the presence of cardiometabolic multimorbidity on RA features of poor prognosis. The purposeful selection process of covariates started by a univariate analysis of each variable; any variable having a significant univariate test and/or a clinical relevance was selected as a possible candidate for the multivariate logistic regression analysis. Conversely, covariates were removed from the model if they were nonsignificant or without a clinical relevance. At the end of this multistep process of deleting and refitting, the multivariate models were built, and odds ratio (OR) estimations of the possible influence of the presence of cardiometabolic multimorbidity on RA features of poor prognosis were provided. Two-sided *P* values < .05 were considered statistically significant. The Statistics Package for Social Sciences (SPSS version 17.0, SPSS Inc.) was used for all analyses.

## 3. Results

### 3.1. Descriptive statistics

In this evaluation, 757 participants were assessed (female, 56.6%; mean age 64.7 ± 12.3 years) (Table 1); 13.5% of participants showed cardiometabolic multimorbidity. These were older (participants with cardiometabolic multimorbidity: 70.4 ± 9.0 years vs participants without: 63.9 ± 13.1 years, *P* < .001) and characterized by a longer disease duration (participants with cardiometabolic multimorbidity: 9.5 (15) years vs participants without: 6.0 (16) years, *P* = .023). Participants with cardiometabolic multimorbidity were more frequently burdened by an extra-articular disease (participants with cardiometabolic multimorbidity: 17.7% vs participants without: 8.2%, *P* = .029). Particularly, 26 participants were affected by a secondary Sjogren’s syndrome. Interstitial lung disease was identified in 16 participants, whereas 3 had a rheumatoid vasculitis. Finally, 3 participants had rheumatoid nodules. Participants with cardiometabolic multimorbidity displayed smoking habit when compared with those without (participants with cardiometabolic multimorbidity: 50.0% vs participants without: 36.6%, *P* = .003). A lower percentage of participants with cardiometabolic multimorbidity was in clinical remission than others (participants with cardiometabolic multimorbidity: 8.5% vs participants without: 15.7%, *P* = .048). These participants were less frequently treated with methotrexate (participants with cardiometabolic multimorbidity: 65.5% vs participants without: 80.2%, *P* = .009). and with hydroxychloroquine (participants with cardiometabolic multimorbidity: 14.6% vs participants without: 28.4%, *P* = .015). Furthermore, participants with cardiometabolic multimorbidity showed a more frequent history of bDMARD failure (participants with cardiometabolic multimorbidity: 78.4% vs participants without: 40.4%, *P* < .001), due to inefficacy, either primary or secondary, adverse events, contraindications, or intolerance. Finally, participants with cardiometabolic multimorbidity were characterized by a higher prevalence of HBP (participants with cardiometabolic multimorbidity: 85.3% vs participants without: 19.8%, *P* < .001), dyslipidaemia (participants with cardiometabolic multimorbidity: 85.3% vs participants without: 11.4%, *P* < .001), T2D (participants with cardiometabolic multimorbidity: 39.0% vs participants without: 5.1%, *P* < .001), and clinical atherosclerosis (participants with cardiometabolic multimorbidity: 14.6% vs participants without: 1.2%, *P* < .001).

**Table 1:** Demographic and clinical features of the evaluated participants with rheumatoid arthritis.

	Whole cohort	Participants without cardiometabolic multimorbidity	Participants with cardiometabolic multimorbidity	*P* value
Number of Patients, (%)	757 (100%)	655 (86.5%)	102 (13.5%)	/
Demographic characteristics				
Gender (female/male), (%)	56.6/43.4%	73.4/26.6%	36.5/63.4%	<.001
Age, yr, mean ± SD	64.7 ± 12.3	63.9 ± 13.1	70.4 ± 9.0	<.001
BMI, mean ± SD	27.4 ± 5.3	24.9 ± 2.8	28.7 ± 3.9	.634
RA features				
Disease duration at the first observation, yr, median (IQR)	6.0 (12)	6.0 (16)	9.5 (15)	.023
<1 yr, (%)	22.3%	21.2 %	9.7%	.009
Between 1 and 5 years, (%)	25.3%	25.3%	25.6%	.899
Between 5 and 10 years, (%)	52.4%	53.5%	64.7%	.003
RF and/or ACPA, (%)	68.3%	58.9%	60.2%	.750
Extra-articular disease, (%)	6.7%	8.2%	17.7%	.029
Joint surgery, (%)	11.4%	10.4%	9.8%	.463
Smoking habit, (%)	34.4%	36.6%	50.0%	.003
RA disease activity				
DAS28, mean ± SD	4.0 ± 1.6	3.9 ± 1.5	4.2 ± 1.3	.117
High disease activity, (%)	22.0%	21.1%	29.2%	.214
Moderate disease activity, (%)	47.2%	47.4%	46.3%	.917
Low disease activity, (%)	15.8%	15.9%	15.8%	.923
Remission, (%)	14.9%	15.7%	8.5%	.048
RA therapies				
GCs low dose, (%)	78.2%	70.4%	63.5%	.807
MTX, (%)	71.9%	80.2%	65.5%	.009
HCQ, (%)	25.9%	28.4%	14.6%	.015
LEF, (%)	13.1%	15.1%	12.3%	.484
SSZ, (%)	7.1%	6.8%	9.8%	.357
Biologic DMARDs, (%)	43.5%	48.2%	42.6%	.558
TNFis, (%)	26.2%	28.2%	23.1%	.594
Non-TNFis, (%)	17.3%	20.0%	17.1%	.537
History of bDMARD failure (327 RA patients), (%)	38.8%	40.4%	78.4%	<.001
Comorbidities				
HBP, (%)	27.3%	19.8%	85.3%	<.001
Dyslipidaemia, (%)	19.9%	11.4%	85.3%	<.001
T2D, (%)	9.0%	5.1%	39.0%	<.001
Clinical atherosclerosis, (%)	2.7%	1.2%	14.6%	<.001

ACPA = anti-citrullinated protein antibodies, BMI = body mass index, bDMARDs = biologic disease-modifying antirheumatic drugs, DAS28 = 28-joint disease activity score, DMARD = disease-modifying antirheumatic drug, GCs = glucocorticoids, HCQ = hydroxychloroquine, HBP = high blood pressure, IQR = interquartile range, LEF = leflunomide, MTX = methotrexate, RA = rheumatoid arthritis, RF = rheumatoid factor, SD = standard deviation, SSZ = sulfasalazine, T2D = type 2 diabetes, TNFis = tumor necrosis factor inhibitors.

### 3.2. Cardiometabolic multimorbidity as predictor of RA severity features of poor prognosis

In our cohort, regression logistic models, both univariate and multivariate, were built to evaluate the predictive role of cardiometabolic multimorbidity on the likelihood of the presence of selected RA features of poor prognosis (Table 2). Furthermore, the presence of cardiometabolic multimorbidity resulted to be a significant predictor of ACPA positivity in univariate analyses (OR: 1.52, 95% CI: 1.10–2.09, *P* = .009). This finding has been also observed in multivariate analysis (OR: 1.47, 95% CI: 1.06–2.04, *P* = .020), cardiometabolic comorbidity resulted to be a significant predictor of ACPA positivity adding to the model age, gender, and smoking habit. Cardiometabolic multimorbidity also predicted the presence of extra-articular manifestations in univariate analysis (OR: 3.26, 95% CI: 1.77–5.89, *P* < .001). The presence of cardiometabolic multimorbidity resulted to be a negative predictor of being in clinical remission in univariate analysis (OR: 0.53, 95% CI: 0.39–0.97, *P* = .041). Multivariate analysis also showed that cardiometabolic multimorbidity resulted a significant negative predictor of clinical remission (OR: 0.61, 95% CI: 0.41–0.96, *P* = .035) adding to the model age, gender, positivity for RF and ACPA. Finally, participants with cardiometabolic multimorbidity had a higher probability of previous bDMARD failure; these analyses were performed only in participants treated with such drugs. Cardiometabolic multimorbidity were significantly associated with the history of bDMARD failure in univariate analysis (OR: 1.73, 95% CI: 1.24–2.43, *P* < .001). This finding has been also observed in multivariate analysis, cardiometabolic multimorbidity resulted a significant predictor of the history of bDMARD failure (OR: 7.17, 95% CI: 3.61–14.2, *P* < .001), adding to the model age, gender, positivity for RF and ACPA.

**Table 2:** Regression logistic analyses assessing the possible predictive role of cardiometabolic multimorbidity on disease features in participants with rheumatoid arthritis.

Clinical Variables	OR	95% CI	*P* value
ACPA – univariate analysis			
Cardiometabolic multimorbidity	1.52	1.10–2.09	.009
ACPA – multivariate analysis			
Cardiometabolic multimorbidity	1.47	1.06–2.04	.020
Age	0.99	0.99–1.03	.343
Gender	1.12	0.89–1.3	.336
Smoking	1.15	0.93–1.43	.213
Extra-articular manifestations – univariate analysis			
Cardiometabolic multimorbidity	3.26	1.77–5.89	<.001
Extra-articular manifestations – multivariate analysis			
Cardiometabolic multimorbidity	1.73	0.89–3.37	.108
Age	1.02	0.99–1.04	.106
Gender	2.01	1.18–3.43	.010
ACPA	2.06	1.19–3.59	.010
DAS28	0.93	0.77–1.13	.489
Remission – univariate analysis			
Cardiometabolic multimorbidity	0.53	0.39–0.97	.041
Remission – multivariate analysis			
Cardiometabolic multimorbidity	0.61	0.41–0.96	.035
Age	1.01	0.99–1.02	.225
Gender	0.58	0.26–1.30	.172
ACPA	0.88	0.53–1.45	.615
RF	0.88	0.53–1.46	.626
*bDMARD failure – univariate analysis*			
Cardiometabolic multimorbidity	1.73	1.24–2.43	<.001
bDMARD failure – multivariate analysis			
Cardiometabolic multimorbidity	7.17	3.61–14.2	<.001
Age	1.00	0.98–1.022	.790
Gender	3.8	2.30–6.30	.001
ACPA	1.56	0.81–2.99	.176
RF	0.64	0.32–1.25	.643

ACPA = anti-citrullinated protein antibodies, bDMARD = biologic disease-modifying antirheumatic drug, DAS28 = disease activity score including 28 joints, OR = odds ratio, RF = rheumatoid factor.

## 4. Discussion

In this work, disease features of RA participants with cardiometabolic multimorbidity were explored. Older age, longer disease duration, lower frequency of remission, and an increased rate of bDMARD failure were observed in these participants. Furthermore, the presence of such comorbidities resulted to be associated with RA features of poor prognosis including ACPA positivity, extra-articular manifestations, and lack of achievement of clinical remission.

Participants with cardiometabolic multimorbidity were older and with a longer disease duration. The prevalence of these disorders increases with age but also a prolonged exposure to a chronic inflammatory process may influence this issue.^[3,5,8,9]^ In this context, multiple lines of evidence reported an increased incidence of RA in patients aged over 60 years focusing the attention on aging related issues.^[34,35]^ In addition, a longer disease duration may be associated with a chronic use of nonsteroidal anti-inflammatory drugs and/or glucocorticoids contributing to the occurrence of cardiometabolic multimorbidity.^[36]^ Finally, disability, inactivity, and a more sedentary behavior may characterize long-lasting RA because of the chronic joint involvement.^[37]^

Our analyses also pointed out that participants with cardiometabolic multimorbidity were less frequently in clinical remission. In fact, the active rheumatoid inflammatory process may contribute to enhancing the CV burden risk of these patients.^[2–4,8,9]^ Thus, the maintenance of clinical remission should be considered the pivotal goal for RA management.^[38]^ Furthermore, a treat-to-target intervention, aiming at clinical remission, may also reduce the occurrence of cardiometabolic multimorbidity.^[2,36]^ Additionally, the presence of cardiometabolic multimorbidity were associated with ACPA positivity and extra-articular disease manifestations. ACPA positive patients are characterized by a more sustained inflammatory process and experience an enhanced cardiometabolic burden.^[39]^ Moreover, ACPA may increase the pro-thrombotic and pro-oxidative atherogenic immune cell profile associated with atherosclerosis, and plaque instability.^[40]^ The association between cardiometabolic multimorbidity and extra-articular manifestations may be similarly related to the presence of a prolonged inflammatory state which is involved in the development of both.^[41]^ Therefore, all things considered, our participants with cardiometabolic multimorbidity may possibly have a more severe disease and may be considered as “difficult-to-treat” RA, as recently proposed.^[42]^ The latter is defined as the persistency of signs and/or symptoms suggestive of inflammatory RA disease activity, despite prior treatment with conventional synthetic disease-modifying antirheumatic drugs and at least 2 bDMARDs.^[42,43]^ In this regard, multiple lines of evidence investigated the clinical manifestations of “difficult to treat RA,” mainly assessing specific disease features related to RA, including autoantibodies, extra-articular features, and inflammatory markers.^[44,45]^ However, an international recent survey demonstrated that rheumatology experts considered some cardiometabolic comorbidities as additional determinants of the development of “difficult to treat RA.”^[46]^ Specifically, we assessed this cardiometabolic multimorbidity in identifying a further possible subset of “difficult to treat RA.” Although there is a well-known association between cardiovascular burden and RA,^[2–4,8,9]^ there are multiple lines of evidence that show how these comorbidities are largely unrecognized and underdiagnosed.^[47,48]^ Thus, it is possible that these comorbidities may negatively contribute to RA outcome, and these could be associated with specific clinical manifestations. Consequently, taking together these observations, patients with cardiometabolic multimorbidity may be more difficultly treated.^[42]^ In this regard, our participants with cardiometabolic multimorbidity showed a higher frequency of having a history of either conventional synthetic - and/or bDMARD failure. Many features may complicate the management of these patients with comorbidities, such as a persistent inflammatory activity due to resistance of disease to DMARDs, limited drug options due to adverse drug reactions, and treatment nonadherence.^[42,43]^ Taking together, the presence of cardiometabolic multimorbidity would be a clinical challenge for rheumatologists complicating the practical therapeutic strategy of RA. In fact, we derived that patients with cardiometabolic multimorbidity has an increased probability to have ACPA positivity, extra-articular manifestations, and a history of bDMARD failure. In addition, these patients were less probably in clinical remission according to our data. Thus, it is well known that RA disease severity is usually associated with unfavorable metabolic conditions, but it is also possible that cardiometabolic multimorbidity may be associated with some RA clinical features of poor prognosis.

The observation that cardiometabolic multimorbidity may be associated with some RA clinical features of poor prognosis may be of relevance, highlighting the importance of assessing CV risk to improve long-term outcomes of these patients.^[49,50]^ Our participants with cardiometabolic multimorbidity were more frequently affected by clinical atherosclerosis, confirming their increased CV burden. In fact, despite the improved long-term joint damage due to recent therapeutic strategies,^[38]^ a close relationship between RA and accelerated atherosclerosis is reported, in increasing the morbidity and the mortality of these patients.^[51,52]^ This typical clinical phenotype results from the interaction between the “traditional” CV risk factors and the rheumatoid inflammatory process.^[3,9,53]^ “Traditional” CV risk factors are more prevalent in RA than general population and these could be also underdiagnosed and undertreated,^[5,54,55]^ despite a number of biomarkers has been proposed.^[56–58]^ Furthermore, the rheumatoid inflammatory process may contribute to the pathogenesis of atherosclerotic disease.^[8,9]^ Interestingly, prolonged periods of uncontrolled disease activity, underlying an active pro-inflammatory process, may represent a strong contributor to cardiometabolic risk in RA patients.^[4,59–61]^ This appears to be independent from RA disease duration.^[62]^ In this context, the importance of reaching and maintaining the clinical remission is also pointed out by multiple lines of evidence since it is associated with a lesser risk of having these comorbidities.^[63–65]^ Intriguingly, a recent body of evidence has suggested the usefulness of therapeutic strategies designed for RA in the management of cardiometabolic comorbidities.^[65–67]^ In previous experiences, the role of methotrexate and hydroxychloroquine has been proposed in reducing the cardiometabolic burden of these patients.^[68–71]^ More recently, the cardiometabolic benefits of the administration of some bDMARDs, mainly IL-1 and IL-6 inhibitors, has been reported in managing patients with RA affected by cardiometabolic comorbidity.^[72–76]^

Although providing an evaluation of the influence of cardiometabolic multimorbidity in RA, this study is affected by different limitations due to the observational design, limiting the generalization of the results and the external validity. In addition, considering the cross-sectional design of our study, we did not make a causal inference. Moreover, we used clinical features of RA poor prognosis which were available in our “real-life” clinical practice; more accurate markers should be evaluated in future specific designed studies. In our study, we did not evaluate the possible differential influence of cardiometabolic multimorbidity on RA features when compared with fully defined metabolic syndrome. In this context, it must be pointed out that insights on clustering patterns in RA are limited to the metabolic syndrome,^[27]^ suggesting additional data on broader cardiovascular risk factor clusters. A further limitation of our study may be associated to the lack of stratification of bDMARD failure according to different timepoints and reasons of previous discontinuation. However, the present study has not specifically developed to evaluate the efficacy of any drug and further studies are needed to entirely clarify the influence of cardiometabolic multimorbidity on bDMARD efficacy. Therefore, taking together these limitations, further studies are needed to fully elucidate this issue.

## 5. Conclusion

In conclusion, clinical features of RA participants with cardiometabolic multimorbidity were described, identifying a possible disease subset to be considered as “difficult-to-treat.” These comorbidities may also suggest the need of a new management approach since the treatment goal is more difficulty achieved in those patients. Further specific designed and adequately powered studies are needed to fully elucidate this issue and the influence of cardiometabolic multimorbidity on RA clinical scenario. Moreover, additional data are warranted to compare the possible differential influence of cardiometabolic multimorbidity on RA features over time when compared with fully defined metabolic syndrome.

## Acknowledgements

The authors thank Mrs. Federica Sensini for her technical assistance.

## Author contributions

**Conceptualization:** Piero Ruscitti, Claudia Di Muzio, Alessandro Conforti.

**Data curation:** Piero Ruscitti, Claudia Di Muzio, Alessandro Conforti, Ilenia Di Cola, Viktoriya Pavlych, Luca Navarini, Damiano Currado, Alice Biaggi, Stefano Di Donato, Annalisa Marino, Sebastiano Lorusso, Francesco Ursini, Roberto Giacomelli, Paola Cipriani.

**Formal analysis:** Piero Ruscitti, Claudia Di Muzio, Alessandro Conforti, Ilenia Di Cola, Viktoriya Pavlych, Luca Navarini, Damiano Currado, Alice Biaggi, Stefano Di Donato, Annalisa Marino, Sebastiano Lorusso, Francesco Ursini, Roberto Giacomelli, Paola Cipriani.

**Investigation:** Piero Ruscitti, Claudia Di Muzio, Alessandro Conforti, Ilenia Di Cola, Viktoriya Pavlych, Luca Navarini, Damiano Currado, Alice Biaggi, Stefano Di Donato, Annalisa Marino, Sebastiano Lorusso, Francesco Ursini, Roberto Giacomelli, Paola Cipriani.

**Methodology:** Piero Ruscitti, Claudia Di Muzio, Alessandro Conforti, Ilenia Di Cola, Viktoriya Pavlych, Luca Navarini, Damiano Currado, Alice Biaggi, Stefano Di Donato, Annalisa Marino, Sebastiano Lorusso, Francesco Ursini, Roberto Giacomelli, Paola Cipriani.

**Project administration:** Piero Ruscitti, Paola Cipriani.

**Resources:** Piero Ruscitti, Roberto Giacomelli, Paola Cipriani.

**Software:** Piero Ruscitti, Claudia Di Muzio, Alessandro Conforti, Ilenia Di Cola, Viktoriya Pavlych, Luca Navarini, Damiano Currado, Alice Biaggi, Stefano Di Donato, Annalisa Marino, Sebastiano Lorusso, Francesco Ursini, Roberto Giacomelli, Paola Cipriani.

**Supervision:** Piero Ruscitti, Francesco Ursini, Roberto Giacomelli, Paola Cipriani.

**Validation:** Piero Ruscitti, Claudia Di Muzio, Alessandro Conforti, Ilenia Di Cola, Viktoriya Pavlych, Luca Navarini, Damiano Currado, Alice Biaggi, Stefano Di Donato, Annalisa Marino, Sebastiano Lorusso, Francesco Ursini, Roberto Giacomelli, Paola Cipriani.

**Visualization:** Piero Ruscitti, Claudia Di Muzio, Alessandro Conforti, Ilenia Di Cola, Viktoriya Pavlych, Luca Navarini, Damiano Currado, Alice Biaggi, Stefano Di Donato, Annalisa Marino, Sebastiano Lorusso, Francesco Ursini, Roberto Giacomelli, Paola Cipriani.

**Writing – original draft:** Piero Ruscitti, Claudia Di Muzio, Alessandro Conforti.

**Writing – review & editing:** Piero Ruscitti, Claudia Di Muzio, Alessandro Conforti, Ilenia Di Cola, Viktoriya Pavlych, Luca Navarini, Damiano Currado, Alice Biaggi, Stefano Di Donato, Annalisa Marino, Sebastiano Lorusso, Francesco Ursini, Roberto Giacomelli, Paola Cipriani.
